# Palmitoylation in focus: bridging reproductive biology and translational research

**DOI:** 10.1186/s40659-025-00601-w

**Published:** 2025-04-28

**Authors:** Suriyaraj Shanmugasundaram Prema, Deepankumar Shanmugamprema

**Affiliations:** 1 Department of Biotechnology, Rathinam Technical Campus, Rathinam Techzone, Pollachi Rd, 641021 Eachanari, Coimbatore, Tamil Nadu India; 2 Department of Research Analytics, Saveetha Institute of Medical and Technical Sciences, Saveetha Dental College and Hospitals, Saveetha University, Chennai, 600077 India

**Keywords:** Protein palmitoylation, Sperm motility, Protein tyrosine phosphorylation, Calcium, Reactive oxygen species

Protein palmitoylation, a reversible post-translational modification, plays a significant role in regulating cellular dynamics, influencing processes such as membrane localization, signal transduction, and protein stability. A recent study by Xiong et al. (2025) [1] titled *“Protein Palmitoylation is Involved in Regulating Mouse Sperm Motility via Calcium*,* Protein Tyrosine Phosphorylation*,* and Reactive Oxygen Species”*, published in *Biological Research* captured our attention. By examining the balance between palmitoylation and depalmitoylation, this study provides an important framework for understanding its potential as a therapeutic target in conditions like cancer, neurodegenerative disorders, and immune dysfunction. Notably, this study provides novel insights into the in situ localization of protein palmitoylation in sperm, shedding light on its critical role in sperm motility and potential underlying mechanisms. The study investigates the interplay between palmitoylation, calcium signaling, protein phosphorylation, and reactive oxygen species (ROS) through methodologies including click chemistry (a set of fast, highly specific reactions for labeling and detecting biomolecules) and computer-assisted sperm analysis (CASA), an automated method for quantifying sperm motility. These findings contribute to a deeper understanding of sperm physiology and suggest potential applications for treating male infertility. Given the broader implications of this research, this letter seeks to further the discussion by incorporating recent advancements, addressing key limitations, and suggesting future directions for translational applications. Protein palmitoylation presents a promising avenue for uncovering novel pathways for therapeutic interventions across a wide range of diseases.

Palmitoylation, characterized by the reversible attachment of palmitic acid to cysteine residues of target proteins, plays a vital role in regulating protein localization, stability, and function. As highlighted in prior research, this dynamic lipidation modulates signal transduction in immune and tumor cells by affecting protein interactions and compartmentalization [2, 3]. The reversible nature of this lipid modification allows for precise control of protein activity, akin to other dynamic post-translational modifications like phosphorylation. Xiong et al.‘s identification of palmitoylated proteins such as heat shock protein 90 (HSP90) further supports the regulatory role of palmitoylation in sperm motility. HSP90 has been implicated in a variety of cellular processes, including protein folding and stress response, making it a critical target for further investigation. The observed disruption of sperm motility upon inhibition of palmitoylation with 2-bromopalmitic acid (2BP) demonstrates the essential role of this modification in maintaining normal reproductive functions. These findings resonate with earlier reports on palmitoylation’s regulatory influence on cellular signaling pathways, including immune activation and tumor suppression [4].

While Xiong et al. focus on reproductive biology, similar regulatory mechanisms of palmitoylation extend into other fields such as oncology, immunology, and neurodegeneration. In cancer biology, palmitoylation plays a critical role in modulating oncogenic signaling networks. For instance, studies have shown that blocking epidermal growth factor receptor (EGFR) palmitoylation, through DHHC20 knockdown or expression of a palmitoylation-resistant EGFR mutant, alters downstream signaling in *KRAS*-mutant non-small cell lung cancer (NSCLC). This inhibition reduces PI3K activation, MYC abundance, and tumor proliferation, while also increasing sensitivity to PI3K inhibitors [5]. Given these insights, targeting DHHC20 to disrupt EGFR palmitoylation presents a promising therapeutic strategy for KRAS-mutant cancers, potentially overcoming resistance to current treatments. Additionally, understanding how external conditions shape palmitoylation in vivo could provide valuable insights into disease mechanisms and therapeutic interventions. Advancements in proteomic technologies for palmitoylome profiling will be instrumental in identifying novel biomarkers and drug targets, ultimately facilitating precision medicine approaches tailored to palmitoylation-related pathologies. Xiong et al.’s study highlights the interplay between palmitoylation and calcium signaling, consistent with findings in cardiovascular and neurological systems. In the cardiovascular system, palmitoylation modulates voltage-gated calcium channels, impacting heart function and arrhythmias [6]. Similarly, in the nervous system, it influences neurotransmission by regulating NMDA receptors and synaptic plasticity. Disruptions in this crosstalk are linked to diseases such as cardiac arrhythmias and neurodegenerative disorders, making palmitoylation a potential therapeutic target. Understanding how palmitoylation orchestrates calcium signaling in these systems could open new avenues for therapeutic interventions, such as targeting palmitoyltransferases (DHHC enzymes) to modulate calcium-dependent processes in disease states.

The methodological approach adopted by Xiong et al., which includes the use of click chemistry and computer-assisted sperm analysis (CASA), represents a robust and innovative means of visualizing and quantifying palmitoylation in real-time. Click chemistry offers a high degree of specificity and sensitivity, making it an invaluable tool for studying dynamic lipid modifications. CASA, on the other hand, provides quantitative insights into sperm motility, offering a comprehensive understanding of how palmitoylation influences functional outcomes [7]. However, expanding this analysis through advanced proteomics techniques, such as mass spectrometry, could enable the identification of additional palmitoylated proteins, thereby offering a more holistic view of palmitoylation dynamics. Furthermore, while the authors effectively delineate the acute effects of 2BP on sperm motility, long-term studies are warranted to explore potential compensatory mechanisms that may arise from chronic palmitoylation inhibition. These studies could provide critical insights into the broader physiological implications of manipulating palmitoylation pathways.

Expanding the study to include human sperm samples is essential for translating these findings to clinical applications. Given the documented interspecies differences in sperm signaling pathways, it remains uncertain whether the regulatory mechanisms observed in mice are conserved in humans. Comparative studies could bridge this knowledge gap and enhance the translational potential of the findings. Investigating the interplay between palmitoylation and other post-translational modifications, such as ubiquitination and phosphorylation, could reveal synergistic regulatory mechanisms essential for sperm function. The integration of multi-omics approaches including proteomics, transcriptomics and metabolomics could provide a more comprehensive understanding of these interactions. Exploring the effects of metabolic and oxidative stress on palmitoylation in reproductive tissues may shed light on how environmental factors impact male fertility. For instance, understanding the role of ROS in modulating palmitoylation patterns could inform the development of antioxidant-based therapeutic strategies. The development of small-molecule modulators targeting palmitoylation pathways offers an exciting avenue for treating male infertility. For example, fine-tuning the enzymes responsible for palmitoylation using selective small-molecule agents may help restore the normal balance of protein modifications in sperm cells, thereby improving motility and overall fertility. This targeted approach could be integrated with current treatment strategies. Additionally, integrating antioxidant therapies with palmitoylation inhibitors may mitigate ROS-induced sperm dysfunction. Such combination therapies could provide a dual approach to enhancing sperm motility and viability.

In conclusion, the work of Xiong et al. represents a noteworthy development in understanding the molecular underpinnings of sperm motility. By contextualizing these findings within broader research on palmitoylation, we can appreciate its vast regulatory potential across biological systems. Continued research into this dynamic modification will undoubtedly yield transformative insights with implications beyond reproductive health, extending into oncology, neurobiology, and immunology.

## Data Availability

The datasets analysed during the study are available from the corresponding author upon reasonable request.

## Abbreviations

ROS: Reactive Oxygen Species
HSP90: Heat Shock Protein 90
2BP: 2–Bromopalmitic Acid
EGFR: Epidermal Growth Factor Receptor
NSCLC: Non–Small Cell Lung Cancer
PI3K: Phosphoinositide 3–Kinase
MYC: MYC Proto–Oncogene
DHHC: Asp–His–His–Cys (Palmitoyltransferase Enzyme Family)
NMDA: N–Methyl–D–Aspartate
CASA: Computer–Assisted Sperm Analysis
